# Translation and cultural adaption of MacLeod Clark professional identity scale among Chinese therapy students

**DOI:** 10.1371/journal.pone.0318101

**Published:** 2025-01-28

**Authors:** Xiaoyi Shu, Chun Feng, Chak-Lam Ip, Xin Zhang, Nan Yang, Shibo Li, Jia Han, Weibing Wu, Alec Knight

**Affiliations:** 1 Department of Physiotherapy, National Hospital for Neurology and Neurosurgery, University College London NHS Foundation Trust, London, United Kingdom; 2 The Centre of Rehabilitation Therapy, The First Rehabilitation Hospital of Shanghai, Rehabilitation Hospital Affiliated to Tongji University, Shanghai, China; 3 Department of Stroke Medicine, University College London NHS Foundation Trust, London, United Kingdom; 4 Department of Rehabilitation, School of Medicine, Tongji University, Shanghai, China; 5 School of Journalism and Communication, Shanghai University of Sport, Shanghai, China; 6 School of Rehabilitation Science, Hubei University of Chinese Medicine, He Bei, China; 7 College of Rehabilitation Sciences, Shanghai University of Medicine and Health Sciences, Shanghai, China; 8 School of Kinesiology, Shanghai University of Sport, Shanghai, China; 9 Faculty of Life Sciences & Medicine, King’s College London, London, United Kingdom; The World Islamic Sciences and Education University, JORDAN

## Abstract

**Background:**

Fostering a strong professional identity (PI) enhances career fulfillment. In China, therapy education is undergoing development, integrating both Western and traditional health concepts, causing inconsistent PI among therapy students. To date, no validated tools exist to measure and monitor PI of Chinese therapy students. This study aimed to translate and validate the 9-item MacLeod Clark Professional Identity Scale (MCPIS-9) for this purpose.

**Design:**

This study involved translation and cultural adaptation of the MCPIS-9, followed by a rigorous assessment of its model fit and psychometric properties using data collected via an online questionnaire.

**Methods:**

A forward- and backward- translation process was conducted. Content validity was evaluated using item-level content validity index (I-CVI) and scale level content validity index average method (S-CVI/Ave). Therapy students across all grades at undergraduate and postgraduate levels in China were eligible. Exploratory factor analysis (EFA) examined the underlying factor structure. Model fit was evaluated through confirmatory factor analysis (CFA) using the Comparative Fit Index (CFI), Tucker-Lewis Index (TLI), Standardized Root Mean Square Residual (SRMR) and Root Mean Square of Error of Approximation (RMSEA). Convergent validity was assessed through Pearson’s correlations coefficient (r) with the Professional Identity Scale for Health Students and Professionals (PISHSP). Internal consistency was examined using Cronbach’s Alpha (Cα) and McDonald’s Omega (ω).

**Results:**

A total of 1054 students participated. Content validity was excellent (I-CVI = 0.86–1.0, S-CVI/Ave = 0.98). EFA indicated a two-factor structure with acceptable model fit (CFI = 0.978; TLI = 0.968; SRMR = 0.033; RMSEA = 0.063). Reliability was strong (Cα = 0.835; ω = 0.817). Convergent validity demonstrated a strong correlation (r = 0.75) with the PISHSP.

**Conclusions:**

The Chinese MCPIS-9 is a reliable and valid tool for assessing PI among therapy students. Future research could focus on refining item 4 of this tool, potentially through further exploration of therapy students’ perceptions of PI within the unique context of the Chinese healthcare system.

## Introduction

Professional identity (PI) refers to attitudes, values, knowledge, beliefs and skills shared with others within professional group [1]. A stronger PI is associated with improved work performance, greater job satisfaction and higher retention rater [2]. Healthcare professionals with a stronger PI have also been found to have a positive influence on their co-workers and patients [3, 4].

The integration of PI into healthcare education is believed to enhance a sense of belonging and resilience [5]. However, there is no consensus regarding the structure of PI. A systematic review of PI during internships identified 10 components [6]. Consequently, scales assessing PI for health and social care students vary in their components and lengths [1, 7, 8]. Belonging is one of the most frequently assessed elements of PI [1, 5, 8]. Based on current understanding of PI, leaders of newly developed professional programmes could prepare their students by developing not only knowledge and skills, but also resilience, sense of belonging and job satisfaction.

In China, modernization of therapy education started in the early-1990s, and experienced rapid development following a significant earthquake in 2008 [9]. To date, only six physiotherapy programmes across the whole country are accredited by the World Confederation of Physical Therapy [10]. The registered professional title in China is Rehabilitation Therapist (RT) [11]. Curricula for RT education vary greatly, with both Western and traditional Chinese therapies co-existing in most RT programmes in varying proportions [12, 13]. A wide range of disciplines, including physiotherapy, occupational therapy, and speech and language therapy are often incorporated into a single RT programme [14]. The complexity of RT education makes it difficult to form a unified PI among students. Therefore, PI may serve as an indicator of successful therapy education as RT education continues to evolve.

Most PI scales have been designed in English for medical and nursing students [1, 15, 16]. The Professional Identity Scale for Health Students and Professionals (PISHSP) is a 33-item scale, originally created in Mandarin in Taiwan and later translated into English for publication [8]. This generic scale comprises four components and was primarily validated with qualified health professionals [8]. Despite demonstrating good reliability, and construct, convergent and discriminant validity, this tool has not been specifically tested among therapy students [8], and its length may negatively impact response and completion rates [17].

The 9-item MacLeod Clark Professional Identity Scale (MCPIS-9) was initially created to assess PI of first-year health and social care students in pre-registration professional programmes in the UK, including physiotherapy students [1]. This short scale was found to have satisfactory internal consistency, construct validity and predictive validity [1, 15, 16]. However, only 12% of the sample in the initial study were physiotherapy students [1]. Moreover, the scale’s one-component structure, as reported in the initial study, was challenged by Cowin and colleagues, whose analysis suggested a three-factor model [1, 15, 16]. Therefore, we conducted this study to 1) translate and culturally adapt the MCPIS-9 for therapy students across all grades and levels in China, 2) test the goodness of model fit and examine the psychometric properties of the translated MCPIS-9 and 3) make recommendations for the use and further development of the translated version of the MCPIS-9.

## Material and method

### Study design

This study was approved by the Committee for Ethics in Human Research at Shanghai University of Sport (102772022RT038). It employed a multi-stage design encompassing the translation and cultural adaptation of the MCPIS-9, followed by model fit and psychometric testing using data from an online questionnaire completed by Chinese therapy students. The translation process included forward and backward translation, expert review, and pilot testing to ensure cultural and linguistic suitability. Psychometric testing evaluated the reliability and validity of the translated MCPIS-9.

### Instrument

Adam and colleagues [1] developed an instrument assessing PI, comprising four dimensions: namely a professional identity scale (9 items), a team scale (10 items), a cognitive flexibility scale (6 items) and a single item assessing self-reported knowledge. Response options followed a five-point Likert scale, with the following options: strongly disagree (5), disagree (4), neither agree nor disagree (3), agree (2), and strongly agree (1). Subsequent researchers have focused on the first dimension (MCPIS-9) as a shorter version of the instrument, utilising the same Likert rating scale [15, 16].

### Stage 1. Translation and cross-cultural adaptation process

**Fig 1:**
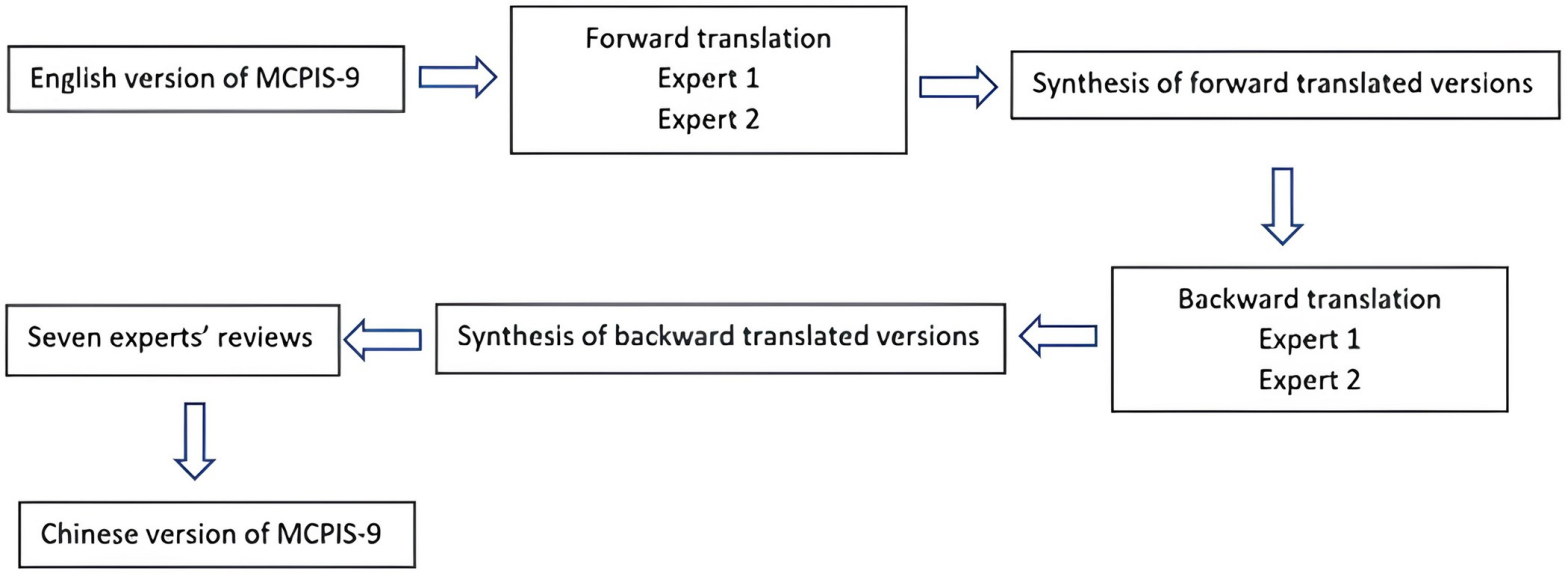
Stage 1- translation process.

The copyright holder granted permission for the translation of the MCPIS-9 from English to Chinese. A standard forward- and backward-translation procedure was conducted, as shown in Fig 1.

#### Forward translation and synthesis

Two Chinese-speaking specialists with relevant expertise independently translated the tool using a structured translation template. One translator, an experienced physiotherapist, currently works as a therapy manager in the U.K. The second translator, holding a Master’s degree from the U.K., currently works in a sports rehabilitation department in a university in China. The two translated versions were then reviewed by the research team to resolve any ambiguities or discrepancies. The core research team, consisting of a clinical educator in therapy, a neurophysiotherapy lecturer, and a health science professor, collaboratively developed a synthesized version.

#### Backward translation and synthesis

To ensure independent translation, neither of the translators had access to the original version of the MCPIS-9. The first translator holds a doctoral degree in Physiotherapy from the U.S. and currently serves as a therapy manager in a rehabilitation hospital in Shanghai. The second translator is an associate professor of English literature at a university in China. The two translated versions were reviewed by the core research team to develop a synthesized version. The discussion continued until a minimum of 95% sematic consistency with the original version was achieved.

#### Cultural adaptation

Experts were selected based on the following criteria: (1) holing a Master’s degree or higher in a therapy-related field, and (2) a minimum of three years’ experience as a clinical educator or teaching physiotherapy in a university in China. Seven experts participated in the tool evaluation, providing feedback that informed the cultural adaptation of several items. For example, the translation of the term ‘profession’ was revised from a general concept (行业) to a more specific one (职业) to enhance personal relevance for respondents. One expert suggested replacing items 4 and 9 with three new items. However, due to a lack of robust justification, only minor wording revisions were implemented with the consensus of the core research team.

#### Content validity of the translated MCPIS-9

A panel of seven experts evaluated the content validity of the culturally adapted instrument. The experts assessed the relevance of each item to the target construct using a 4-point scale (1 = not relevant; 2 = somewhat relevant; 3 = quite relevant; 4 = highly relevant) [18]. Content validity was tested using the item-level content validity index (I-CVI) and scale-level content validity index (S-CVI). An I-CVI value of 0.78 or higher was considered excellent content validity at the item level [19, 20]. Both the S-CVI universal agreement (S-CVI/UA) and average method (S-CVI/Ave) were considered as high if their values were 0.80 or above [20, 21].

#### Sampling procedure

*Pilot group*. To adhere to best practices, the finalised MCPIS-9 was disseminated to a cohort of undergraduate therapy students from mixed grades (n = 38) at Shanghai University of Sport. Face validity was tested using two questions: one evaluating the clarity of the instructions and the other assessing the adequacy of the tool in measuring PI. Participants were also encouraged to comment on the clarity of each item, leading to minor wording revisions. For example, the translations of ‘connection’ in item 2 and ‘excuse’ in item 4 were adjusted according to participant feedback.

*Main study*. The first author contacted the Admissions Offices of universities and colleges offering therapy programmes across four provinces in China. An explanatory document was sent to teaching assistants, who later disseminated the questionnaire link to students via WeChat, a widely used communication application in China. Nine universities and colleges took part in the study. Inclusion criteria were as follows: therapy students (1) from all academic years, and (2) enrolled in an undergraduate or postgraduate therapy programme eligible for national therapy professional registration on completion. Exclusion criteria included therapy students: (1) who had completed studies and left the university, (2) enrolled in a therapy or related programme not eligible for national therapy professional registration, or (3) who were unable to read or write in Mandarin.

### Stage 2. Goodness of model fit

#### Data collection

Data were collected via an online questionnaire comprising three parts as shown in S1 File. Part one consisted of demographic information, including gender, age group, type of education institute, and study year. Part two contained the translated MCPIS-9 and PISHSP, totaling 42 items. Part three included additional questions related to career choices. Students provided informed consent by ticking the consent item and submitting the completed questionnaire on a polling website (www.wjx.com). Only fully completed questionnaires were saved and reviewed by the authors. Data collection took place between 1^st^ October 2022 and 31^st^ January 2023. Data were stored on encrypted hardware and only shared by author XS with co-authors AK, CF and CI.

#### Descriptive statistics

Statistical analyses were performed using Microsoft Excel (2019), SPSS (version 28.0) and AMOS (version 28.0). The variability of items was assessed using median, standard deviation, skew, kurtosis and corrected item total correlation. Values of skewness and kurtosis between -2 and 2 suggested that the data were approximately normally distributed [22].

#### Model fit assessment

Previous studies reported varying numbers of factors for the tool, with some identifying a one-factor structure [1, 16], while one paper reported a three-factor structure [15]. To address these discrepancies and ensure robust findings, we conducted an exploratory factor analysis (EFA) first to investigate the structure of the translated tool, followed by a confirmatory factor analysis (CFA) to verify the best model fit.

Sample splitting is commonly applied in cross-validating structural equation modelling, particularly for confirming a CFA model [23]. This approach helps to prevent overfitting and improve the generalizability and robustness of the model [23]. Thereby, the total sample was randomly split into two equal datasets of 527 subjects each, allocated for EFA and CFA respectively. Analyses were conducted using SPSS for EFA and AMOS for CFA [24].

Given the normality of data distribution, as indicated by the acceptable values of skewness and kurtosis, a maximum likelihood (ML) extraction method with a Promax rotation was selected for EFA. [25]. The primary run of EFA extracted two factors, differing from findings reported in the previous studies [1, 15, 16]. To explore this discrepancy and enhance the credibility of the findings, EFA was repeated with fixed numbers of factors (1,2,3) for comparative analysis. The number of factors was determined by assessing the scree plot and the cumulative percentage of variance in the initial eigenvalue (≥60%) [26, 27].

Sample adequacy was determined by the Kaiser-Meyer-Olkin (KMO) and Bartlett’s test of sphericity [26]. A KMO value between 0.8 and 1.0 indicated an adequate sample and fit for further analysis [28]. A significant value of the Bartlett’s test of sphericity (p<0.05) indicated that variables were interrelated [28].

The model structure was further confirmed through CFA, in which all three models were tested to determine the best-fitting model. Relative (normed) chi square statistics (X^2^/df) below 5 indicated an acceptable fit, while values below 3 indicated a good fit [29]. Other indicators were also examined for goodness of fit, including the following: (1) comparative fit index (CFI) value ≥ 0.9, (2) Tucker-Lewis index (TLI) value ≥0.95, (3) root mean square error of approximation (RMSEA) value ≤ 0.08, and (4) standardized root mean square residual (SRMR) value ≤ 0.05 [30–32].

### Stage 3. Psychometric property assessment

Construct validity refers to the degree to which a scale accurately assesses the construct it is designed to measure [33, 34]. Within this context, convergent validity is the extent to which the same construct is measured using different variables [34]. Convergent validity was assessed using two methods. First, the correlation between the total scores of the MCPIS-9 and the PISHSP was tested using Pearson’s correlation coefficient (r). Scores on all negative items in both scales were reversed prior to the test. The correlation coefficient was interpreted as follows: weak (0.10–0.39), moderate (0.40–0.69), strong (0.70–0.89) and very strong (> 0.89) correlations [20]. Second, factor loadings and average variance extracted (AVE) were calculated to further test convergent validity [35]. Factor loading ≥0.5 and AVE value ≥0.5 indicated adequate convergent validity [28, 36].

Discriminant validity is another type of construct validity. To establish discriminant validity using Fornell-Larcker’s criteria, the AVE for each construct must be greater than the squared correlation between each pair of factors [37]. Because reporting inter-construct correlations is a common practice in the literature, discriminant validity was established if the square root of the AVE for a construct exceeded the correlation between constructs [38].

Reliability analysis (internal consistency) was assessed using Cronbach’s alpha (Cα) for each construct. In addition, McDonald’ omega (ω) was calculated, as it is based on factor analysis rather than the assumption of essential tau-equivalence [39]. Values of Cα and ω ≥0.7 indicated acceptable reliability coefficients [40].

## Results

Completed questionnaires were returned by 1054 students. Demographic characteristics of the sample are listed in Table 1. The ratio of male-to-female participants was approximately 2:1. Nearly 94% of the sample were below age 24. Almost half of the sample were enrolled in sports universities. Participants in the early years of study (Years 1–3) accounted for almost 70% of the sample. The item features of the MCPIS-9 are listed in Table 2. Skewness and kurtosis values suggested the data were normally distributed.

**Table 1:** Characteristics of study participants (n = 1054).

Variable		N	%
Gender	Male	365	34.63%
	Female	689	65.37%
Age (years)	<20	337	31.97%
	20–24	659	62.52%
	25–29	54	5.12%
	30–34	3	0.29%
	≥35	1	0.09%
Type of institute	Medical school	364	34.53%
	Sports university	522	49.53%
	General university	49	4.65%
	College	119	11.29%
Study year	Undergraduate Year1	245	23.14%
	Undergraduate Year2	239	22.68%
	Undergraduate Year3	247	23.43%
	Undergraduate Year4	162	15.37%
	Postgraduate	161	15.28%

**Table 2:** Descriptive statistics of the translated MCSIP-9.

Item	Mean	Standard deviation	Skewness (SE*)	Kurtosis (SE*)
1. I feel like I am a member of this profession 我觉得我是康复行业中的一员	3.83	0.736	-0.750 (0.075)	1.828 (0.151)
2. I feel I have strong ties with members of this profession 我觉得, 我和康复行业的从业人员有很强的联结和共鸣	3.70	0.755	-0.350 (0.075)	0.616 (0.151)
3. I am often ashamed to admit that I am studying for this profession 我经常羞于承认我在学习专业	2.08	0.964	0.859 (0.075)	0.457 (0.151)
4. I find myself making excuses for belonging to this profession 我发现我会找各种说辞来解释我从事康复工作	2.90	1.037	-0.111 (0.075)	-0.625 (0.151)
5. I try to hide that I am studying to be part of this profession 我试图隐瞒正在学习康复专业, 成为这个行业的一员	2.03	0.877	0.785 (0.075)	0.621 (0.151)
6.I am pleased to belong to this profession 从事康复工作让我感到高兴	3.52	0.775	0.174 (0.075)	0.576 (0.151)
7. I can identify positively with members of this profession 我对于康复专业的从业人员有积极的认同感	3.84	0.769	-0.515 (0.075)	0.674 (0.151)
8. Being a member of this profession is important to me 作为康复行业的一员对我很重要	3.54	0.815	-0.245 (0.075)	0.264 (0.151)
9. I feel I share characteristics with other members of the profession 我觉得与康复行业的其他从业人员有共同的特征	3.52	0.735	-0.219 (0.075)	0.517 (0.151)

*SE: standard error

### Goodness of model fit

The KMO value was 0.838, indicating adequacy of the sample for further analysis. The result of Bartlett’s test of sphericity was also significant (p<0.001, Chi-square = 2082.788, df:36). EFA extracted one-, two- and three-factor models. Both the scree plot (S1 Fig) and the cumulative percentage of variance (S1 Table) indicated that both two- and three- factor models could be considered suitable. In the one-factor model, the cumulative percentage of variance was 44.564% (S1 Table), falling below the acceptable threshold of ≥60% [26, 27]. In the three-factor model, the cumulative percentage of variance was the highest among three models (73.393% in S1 Table); however, one of the extracted factors contained only two items, potentially compromising its stability (S2 Table) [26]. Therefore, a two-factor model was deemed the most appropriate.

**Fig 2:**
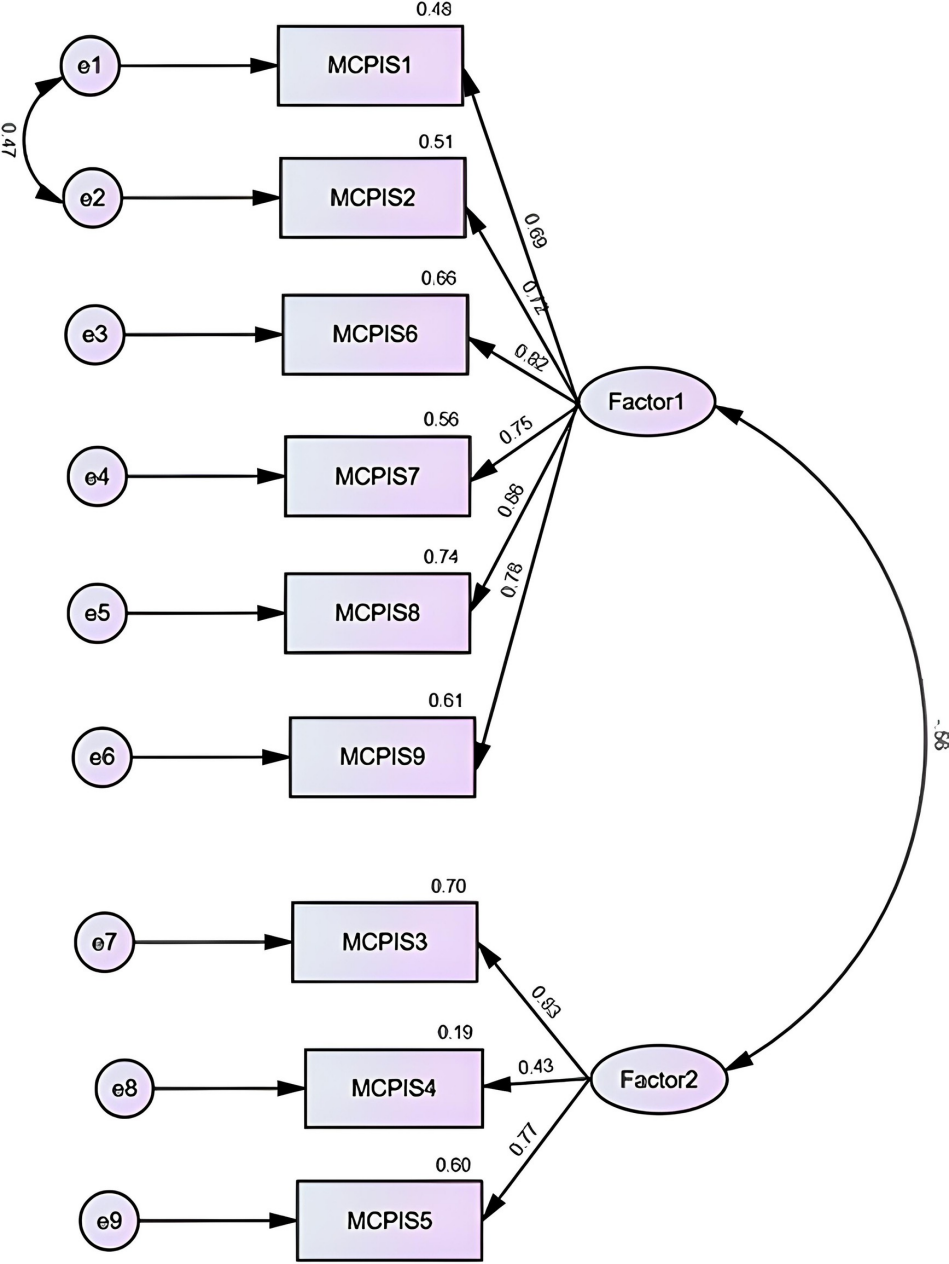
Structure of the two-factor model from CFA.

All three models were further tested using CFA. Table 3 displays the values of the model fit indices. All three models were improved by checking modifications indices. Covariances were added if the modification was greater than 20 and variances were within the same construct [41]. Among the three models, the two- and three-factor models demonstrated similar fit indices, with both showing better overall fit than the one-factor model. The two-factor model marginally outperformed the three-factor model, as indicated by the χ^2^/df and TLI values. The structure of the two-factor model is shown in Fig 2. All factor loadings were close to or above 0.7, except for item 4, which had a loading below 0.5.

**Table 3:** Model fit indices in CFA of the translated MCPIS-9.

Model	X^2^/df^a^	CFI^b^	TLI^c^	RMSEA^d^	SRMR^e^
One-Factor Model	4.008	0.987	0.976	0.076	0.089
Two-Factor Model	3.115	0.978	0.968	0.063	0.033
Three-Factor Model	3.230	0.978	0.967	0.065	0.033

**Abbreviations**:X2/df: relative (normed) chi square statistics; CFI: comparative fit index; TLI: Tucker-Lewis Index; RMSEA: root mean square of error of approximation; SRMR: standardized root mean square residual

**Criterion for a good fit**: a: X^2^/df <5 acceptable, <3 good; b: CFI≥ 0.9; c:TLI ≥0.95; d: RMSEA≤ 0.08; e: SRMR≤ 0.05

In conclusion, the two-factor model proved to be the most optimal, showing the best fit based on both EFA and CFA results. Although the three-factor model explained the most variance and demonstrated similar fit indices to the two-factor model in CFA, it was less stable due to one factor containing only two items.

### Psychometric property assessment

Content validity of the scale was tested using the I-CVI, with values ranging between 0.86 and 1 based on the evaluations of the seven raters, indicating excellent item-level content validity. The S-CVI/Ave (0.98) and the S-CVI/UA (0.89) also suggested excellent scale-level content validity. Detailed content validity measurements are listed in S3 Table.

Convergent validity was first tested using Pearson’s correlation coefficient, which produced a value of 0.75 based on the sums of two scales (p < 0.001). The sum of the MCPIS-9 scores showed moderate to strong correlation to the individual component of the PISHP (*r*: 0.55–0.71), as shown in S4 Table. Table 4 shows the results of the convergent and discriminant validity assessments. The values of the square root of AVE for two factors (0.77 and 0.70 respectively) were greater than the correlation of two factors (0.56). Taken together, these findings provided evidence for both convergent and discriminant validity of the scale.

**Table 4:** Construct validity and reliability measures of the MCPIS-9.

Factor	AVE^a^	√AVE^b^	Factor correlation^c^ Factor 1 Factor 2	Cronbach’s Alpha	McDonald’s Omega
**1 (positive items)**	0.60	0.77	0.56	0.895	0.895
**2 (negative items)**	0.50	0.70	0.56	0.708	0.723
**Total**				0.835	0.817

Abbreviations: a: average variance extracted; b: Square root of average variance extracted; c: Factor correlation from CFA

The analysis suggested satisfactory internal consistency of the MCPIS-9, with Cα and ω values of 0.835 and 0.817 respectively (Table 4). A slight increase in reliability was observed when item 4 was removed, with the overall Cα and ω slightly increasing by 0.027 and 0.033 respectively. Internal consistency for factor 1 (positive items) was high (Cα = 0.895, ω = 0.895), while for factor 2 (reversed negative items) it was acceptable (Cα = 0.708, ω = 0.723).

## Discussion

A strong sense of PI in healthcare education may contribute to build resilience in future careers [5]. With the rapid development of therapy education in China, the consideration and integration of PI among therapy students has become a crucial marker of successful education. PI should be monitored and evaluated across all levels of therapy students. To date, there has been no tool specifically designed or validated for therapy students in China. Though the newly developed PISHSP is a comprehensive tool available in Mandarin, it is a lengthy tool primarily designed for health workers [17]. In addition, translation of a validated tool would allow researchers to test hypotheses in a cross-culturally context [42]. Therefore, the MCPIS-9 was selected for translation in this study. This is a short survey assessing professional belonging within PI among students. Our data analysis reveals the translated version of the MCPIS-9 is a two-factor tool with good validity and reliability. However, further development and analysis are recommended.

### Translation process

In order to achieve linguistic and cultural equivalence [42], some changes of item wording were made during the pilot study according to experts’ and students’ suggestions. One expert recommended removing item 4 and 6 and adding three new items (1-“I do not want to leave this profession”; 2- “I want to make positive changes of this profession through my contribution”; and 3- “I feel frustrated hearing negative news about this profession”). The expert’s recommendations may be attributed to cultural insensitivity during the translation, where the primary effort was made to achieve linguistic equivalence, potentially leading to incongruity of meanings [43]. An additional factor could be metric equivalence [42]. The use of the same words in English and Mandarin may elicit different levels of emotional response. The proposed changes suggested by this particular expert were not incorporated for two reasons. First, the meaning of suggested items 1 and 3 were similar to the reversed version of two existing items in the original tool. Second, the suggested item 2 was classified as the ‘contribution’ element of PI, whereas the MCPIS-9 is a unidimensional tool assessing professional belonging only.

### Goodness of model fit

Previous studies suggested inconsistent findings regarding the factor structure of the MCPIS-9 [1, 15, 16]. Two studies reporting one component applied Principal Component Analysis as an item reduction method [1, 16]. The study reporting three factors had a relatively small sample size of 162 subjects [15]. In contrast, our study, with a larger sample size, assessed one-, two- and three-factor models, and confirmed a two-factor model was the most favorable based on the model fit indices of CFA.

### Validity and reliability assessment

Our study suggested excellent content validity and reliability, and acceptable construct validity of the translated MCPIS-9. This tool not only corelated with the PIHSIP but also showed the strongest correlation to the belonging component of the PISHIP (r = 0.71). Item 4 had the weakest factor loading (0.43), affecting overall validity and reliability. Removing item 4 resulted in a slight improvement in the reliability of the tool, as evidenced by an increase in both Cα and ω values. However, item 4 was retained for two reasons. First, the most affected metrics remained close to acceptable thresholds (i.e., AVE and Cα of factor two). Second, removal of item 4 would leave only two items in a single factor (Fig 2), compromising the stability of the construct [26].

In our experts’ review of the translated MCPIS-9, the majority selected item 4 as ‘highly relevant’ to PI. However, students’ responses revealed a possible difference in understanding of PI. Therefore, further qualitative research may be warranted to explore students’ perspective of PI for future development of this tool. Furthermore, the original tool was developed and tested among pre-registration programme students (Master’s level) [1]. Over 70% of our participants were undergraduate students without clinical experience. This disparity in educational levels may also contribute to inconsistent perceptions of PI. In summary, we recommend retaining item 4 with careful interpretation of the results and possible further development in future studies.

### Strengths and limitations

This study has several notable strengths. To the best of our knowledge, this is the first study translating a PI tool into Chinese. This brief and easily administered tool was specifically validated for therapy students in both English and Mandarin. With the rapid development of therapy in China, this tool could help monitor students’ PI and evaluate the success of modern therapy education. In addition, the inclusion of a large and diverse sample of participants from multiple cities and universities enhances the representativeness therapy students in this study. The substantial sample size boosts the reliability of the results. Finally, the rigorous and robust translation process and comprehensive assessment of the tool provide confidence in the conclusions. This translated tool could also be adapted for other health professional students in future studies.

Nevertheless, this study had several limitations. First, there was a significant gender imbalance in the sample. Only one-third of the sample were male responders, which may limit the generalizability of the results to male therapy students. Second, since this one-off online questionnaire was completed autonomously, test-retest reliability and concurrent validity could not be assessed. Data collected in this study were processed as continuous variables and analyzed using factor analysis in accordance with recommendations [22]. However, some experts argued that polytomous item response theory models should be used to analyze Likert scale data [44].

A systematic review of PI measures reported ‘good’ to ‘excellent’ quality in three previous studies investigating the MCPIS-9 [1, 15, 16], based on the COSMIN checklist [45]. However, the reported results only included the final ratings without providing the details of the rated items. Notably, one study rated as ‘excellent’ failed to enlist a sufficient number of participants for factor analysis and reported incongruent results of EFA and CFA without explaining the causes [15], thereby raising concerns about the reliability of this study’s quality assessment. We conducted a COSMIN cross-cultural validity assessment for our study as presented in S5 Table [46]. While most items received favourable ratings, three items performed less well due to their limited relevance to our sample.

### Implications

There are at least two aspects of this tool requiring further attention. First, item 4 could be further developed due to its negative impact on the overall validity and reliability of the tool. Second, the MCPIS-9 uses a 5-point Likert scale. Tarka [47] suggested a longer response scale (9 or 11 points) could enhance reliability by allowing participants to express opinions more precisely. Future research may explore the feasibility and benefits of implementing a 9 or 11-point Likert scale in the translated MCPIS-9. Furthermore, a Delphi technique could be applied to gain a more culturally accurate understanding of the constitution of PI, providing valuable insights for improving this tool. This translated version of the MCPIS-9 can serve as a valuable tool for researchers examining the level of PI among Chinese-speaking therapy students Ultimately, it could contribute to improving staff retention and advancing therapy education and practice.

## Conclusion

PI among students is often overlooked yet is an essential component of curricula, contributing to resilience and work satisfaction in their future careers. Our study has pioneered PI research within the context of Chinese therapy education. Item 4 was identified as a primary weakness of this translated tool, impacting its validity and reliability. Further developments of item 4 and the establishment of consensus regarding a PI definition among students are recommended for future research. Nevertheless, our findings suggest that the translated MCPIS-9 is a two-factor tool with good validity and reliability.

## Supporting information

S1 Checklist Inclusivity in global research. (DOCX)

S1 File Full version of the questionnaire. (DOCX)

S1 Fig Scree plot of sample exploratory factor analysis (maximum likelihood). (DOCX)

S1 Table Total variance explained tables for one, two and three factor models. (DOCX)

S2 Table Pattern matrix tables for two and three factor models. (DOCX)

S3 Table Content validity measures of the MCPIS-9. (DOCX)

S4 Table Pearson’s correlations coefficient between the MCPIS-9 and the PISHP (total and single construct). (DOCX)

S5 Table Methodological quality assessment using COSMIN checklist. (DOCX)

S1 Data (XLSX)
