# Supplementary material for: Translation and cultural adaption of MacLeod Clark professional identity scale among Chinese therapy students
Source: PLoS One. 2025 Jan 28;20(1):e0318101. doi: 10.1371/journal.pone.0318101 (PMC11774393; doi:10.1371/journal.pone.0318101)
Supplement: S1 File — (DOCX) [file pone.0318101.s002.docx]

**S1** **File: Full version of the questionnaire**

Part 1: Demographics

1. What is your gender?

1.Male 2.Female

1. What is your age?

1.<20 years 2.20-24 years 3.25-29 years 4.30-34 years 5.>=35 years

1. Type of institute you are currently in

1.Medical school 2.Sports university 3.General university College

1. Which year are you in now?

1.Undergraduate Year 1 2.Undergraduate Year 2 3.Undergraduate Year 3 4.Undergraduate Year 4 5.Postgraduate

Part 2: Macleod Clark Professional Identity Scale-9 items

Rating: 1-strongly agree; 2- agree; 3- neither agree nor disagree; 4- disagree; 5- strongly disagree

1. I feel like I am a member of this profession
2. I feel I have strong ties with members of this profession
3. I am often ashamed to admit that I am studying for this profession
4. I find myself making excuses for belonging to this profession
5. I try to hide that I am studying to be part of this profession
6. I am pleased to belong to this profession
7. I can identify positively with members of this profession
8. Being a member of this profession is important to me
9. I feel I share characteristics with other members of the profession

Part 3: Professional Identity Scale for Healthcare students and Professionals

Rating: 1- extremely disagree; 2-disagree very much; 3-moderately disagree; 4- slightly disagree ; 5- neither agree nor disagree; 6- slightly agree; 7- moderately agree; 8- agree very much; 9- extremely agree

Professional Commitment & Devotion

1. Even if the salary is not satisfactory, I (will) remain in healthcare work
2. Even if I am married, I (will) remain in healthcare work
3. I like healthcare work. Even if there are other job opportunities with higher pay, I will not consider them
4. I would regret it if I have to leave healthcare work
5. If I could choose my career again, I would still choose to work in healthcare
6. I (will) devote all my time to healthcare work, even if I have to sacrifice time with my family
7. No matter how many setbacks I may receive in healthcare work, I will never give it up
8. I like my healthcare specialities and am actively preparing myself for the profession
9. My personal goals are highly relevant to my healthcare work
10. I often read journals and books related to healthcare work to enrich my professional knowledge
11. I often think of my healthcare work that I am responsible for right now, as well as the work that I have not yet finished and completed
12. My personality and personal beliefs are consistent with the characteristics and valuesof healthcare work
13. I think healthcare work itself is challenging and stimulating
14. I am always thinking about how to do better in healthcare
15. I believe I can succeed in a healthcare career
16. Engaging in healthcare work gives me psychological satisfaction

Emotional Identification & Belongingness

1. For me, healthcare is the best career that I can do
2. I am proud of being a healthcare professional
3. Even if there are job opportunities other than healthcare, I (will) persist in being a healthcare professional
4. It is important for me to devote myself to healthcare work
5. I have a strong interest in healthcare and always enjoy working in this field
6. I am happy with choosing healthcare as my profession
7. I feel that I am a member of the healthcare profession

Professional Goals & Values

1. I think that healthcare is a professional job
2. I agree with the value of healthcare work
3. I think that healthcare is a respected profession
4. I am sure that the healthcare workers are interested in helping people
5. I believe that healthcare is a profession that can bring certain contributions to the country and society

Self-fulfilment & Retention Tendency

1. I regret choosing healthcare as my career
2. I often want to change my current job
3. If I have the change to choose, under the same working conditions, I will choose a job that is not related to healthcare
4. I have a negative perception of self-worth in response to the current healthcare situation
5. Healthcare is only one of the many jobs I can do, I cannot put my whole heart into it

Part 4: Additional questions

1. Why did you choose to study a therapy programme
2. My personal views on the prospect of rehabilitation industry in China
3. To what degree does the pressure of your current studies affect your career plan. Participants gave a single answer to each item.
