# Supplementary figures and images for: Translation and cultural adaption of MacLeod Clark professional identity scale among Chinese therapy students
Source: PLoS One. 2025 Jan 28;20(1):e0318101. doi: 10.1371/journal.pone.0318101 (PMC11774393; doi:10.1371/journal.pone.0318101)

**S1 Figure: Scree plot of sample exploratory factor analysis (maximum likelihood)**


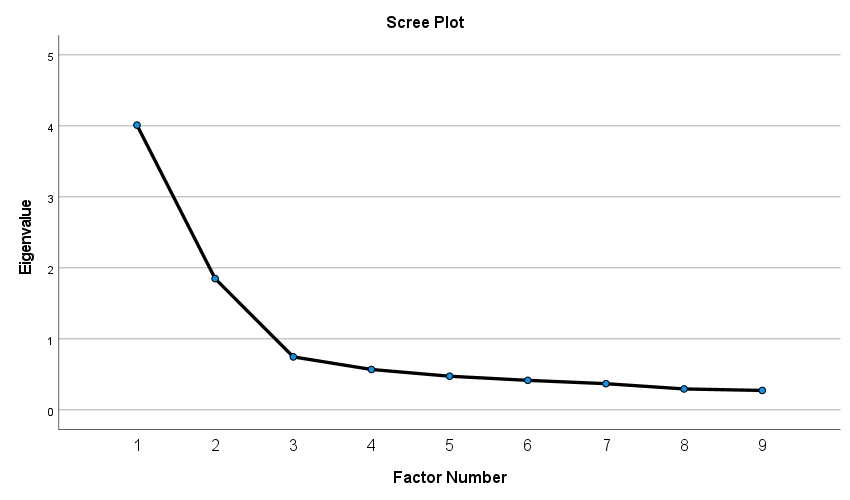

Supplement: S1 Fig — (DOCX) [file pone.0318101.s003.docx]
