# Supplementary material for: Translation and cultural adaption of MacLeod Clark professional identity scale among Chinese therapy students
Source: PLoS One. 2025 Jan 28;20(1):e0318101. doi: 10.1371/journal.pone.0318101 (PMC11774393; doi:10.1371/journal.pone.0318101)
Supplement: S1 Table — (DOCX) [file pone.0318101.s004.docx]

**S1 Tables: Total variance explained tables for one, two and three factor models**

Total variance explained for a one-factor model

| Factor | Initial Eigenvalues | | | Extraction Sums of Squared Loadings | | |
| --- | --- | --- | --- | --- | --- | --- |
|  | Total | % of Variance | Cumulative % | Total | % of Variance | Cumulative % |
| 1 | 4.011 | 44.564 | 44.564 | 3.542 | 39.358 | 39.358 |
| 2 | 1.849 | 20.539 | 65.103 |  |  |  |
| 3 | .746 | 8.290 | 73.393 |  |  |  |
| 4 | .569 | 6.318 | 79.711 |  |  |  |
| 5 | .474 | 5.265 | 84.977 |  |  |  |
| 6 | .416 | 4.618 | 89.595 |  |  |  |
| 7 | .369 | 4.097 | 93.692 |  |  |  |
| 8 | .295 | 3.273 | 96.965 |  |  |  |
| 9 | .273 | 3.035 | 100.000 |  |  |  |
| Extraction Method: Maximum Likelihood. | | | | | | |

Total variance explained for a two-factor model

| Factor | Initial Eigenvalues | | | Extraction Sums of Squared Loadings | | | Rotation Sums of Squared Loadings^a^ |
| --- | --- | --- | --- | --- | --- | --- | --- |
|  | Total | % of Variance | Cumulative % | Total | % of Variance | Cumulative % | Total |
| 1 | 4.011 | 44.564 | 44.564 | 3.593 | 39.928 | 39.928 | 3.522 |
| 2 | 1.849 | 20.539 | 65.103 | 1.404 | 15.602 | 55.530 | 1.671 |
| 3 | .746 | 8.290 | 73.393 |  |  |  |  |
| 4 | .569 | 6.318 | 79.711 |  |  |  |  |
| 5 | .474 | 5.265 | 84.977 |  |  |  |  |
| 6 | .416 | 4.618 | 89.595 |  |  |  |  |
| 7 | .369 | 4.097 | 93.692 |  |  |  |  |
| 8 | .295 | 3.273 | 96.965 |  |  |  |  |
| 9 | .273 | 3.035 | 100.000 |  |  |  |  |
| Extraction Method: Maximum Likelihood. a. When factors are correlated, sums of squared loadings cannot be added to obtain a total variance. | | | | | | | |

Total variance explained for a three-factor model

| Factor | Initial Eigenvalues | | | Extraction Sums of Squared Loadings | | | Rotation Sums of Squared Loadings^a^ |
| --- | --- | --- | --- | --- | --- | --- | --- |
|  | Total | % of Variance | Cumulative % | Total | % of Variance | Cumulative % | Total |
| 1 | 4.011 | 44.564 | 44.564 | 2.720 | 30.225 | 30.225 | 3.467 |
| 2 | 1.849 | 20.539 | 65.103 | 1.783 | 19.811 | 50.036 | 1.680 |
| 3 | .746 | 8.290 | 73.393 | 1.024 | 11.381 | 61.417 | 2.578 |
| 4 | .569 | 6.318 | 79.711 |  |  |  |  |
| 5 | .474 | 5.265 | 84.977 |  |  |  |  |
| 6 | .416 | 4.618 | 89.595 |  |  |  |  |
| 7 | .369 | 4.097 | 93.692 |  |  |  |  |
| 8 | .295 | 3.273 | 96.965 |  |  |  |  |
| 9 | .273 | 3.035 | 100.000 |  |  |  |  |
| Extraction Method: Maximum Likelihood. a.When factors are correlated, sums of squared loadings cannot be added to obtain a total variance. | | | | | | | |
