# Supplementary material for: Translation and cultural adaption of MacLeod Clark professional identity scale among Chinese therapy students
Source: PLoS One. 2025 Jan 28;20(1):e0318101. doi: 10.1371/journal.pone.0318101 (PMC11774393; doi:10.1371/journal.pone.0318101)
Supplement: S2 Table — (DOCX) [file pone.0318101.s005.docx]

**S2 Tables:** **Pattern matrix tables for two and three factor models**

| Pattern matrix for a two-factor model | | |
| --- | --- | --- |
|  | **Factor** | |
|  | **1** | **2** |
| **MCPIS1** | **.726** |  |
| **MCPIS2** | **.761** |  |
| **MCPIS3** |  | **.775** |
| **MCPIS4** |  | **.493** |
| **MCPIS5** |  | **.798** |
| **MCPIS6** | **.761** |  |
| **MCPIS7** | **.701** |  |
| **MCPIS8** | **.831** |  |
| **MCPIS9** | **.730** |  |
| Extraction Method: Maximum Likelihood. | | |
|  | | |
|  | | |

| Pattern matrix for a three-factor model | | | |
| --- | --- | --- | --- |
|  | Factor | | |
|  | 1 | 2 | 3 |
| MCPIS1 | .396 |  | -.401 |
| MCPIS2 |  |  | -.967 |
| MCPIS3 |  | .808 |  |
| MCPIS4 |  | .477 |  |
| MCPIS5 |  | .791 |  |
| MCPIS6 | .760 |  |  |
| MCPIS7 | .739 |  |  |
| MCPIS8 | .876 |  |  |
| MCPIS9 | .704 |  |  |
| Extraction Method: Maximum Likelihood. | | | |
