# Supplementary material for: Translation and cultural adaption of MacLeod Clark professional identity scale among Chinese therapy students
Source: PLoS One. 2025 Jan 28;20(1):e0318101. doi: 10.1371/journal.pone.0318101 (PMC11774393; doi:10.1371/journal.pone.0318101)
Supplement: S3 Table — (DOCX) [file pone.0318101.s006.docx]

**S3 Table: Content validity measures of the MCPIS-9**

| N=7 | 1 | 2 | 3 | 4 | 5 | 6 | 7 | 8 | 9 |
| --- | --- | --- | --- | --- | --- | --- | --- | --- | --- |
| I-CVI | 1 | 1 | 1 | 1 | 1 | 1 | 1 | 1 | 0.86 |
| S-CVI/Ave  S-CVI/UA | 0.98  0.89 | | | | | | | | |

I-CVI: items content validity index; S-CVI/Ave: scale content validity index average; S-CVI/UA: scale content validity index universal agreement
