# Supplementary material for: Translation and cultural adaption of MacLeod Clark professional identity scale among Chinese therapy students
Source: PLoS One. 2025 Jan 28;20(1):e0318101. doi: 10.1371/journal.pone.0318101 (PMC11774393; doi:10.1371/journal.pone.0318101)
Supplement: S4 Table — (DOCX) [file pone.0318101.s007.docx]

**S4: Table Pearson’s correlations coefficient between the MCPIS-9 and the PISHP
(total and single construct)**

|  | | PISHP_SUM_R | PISHP1_SUM | PISHP2_SUM | PISHP3_SUM | PISHP4_SUM_R |
| --- | --- | --- | --- | --- | --- | --- |
| MCPIS_Sum | Pearson Correlation | .753^**^ | .700^**^ | .709^**^ | .554^**^ | .585^**^ |
|  | Sig. (2-tailed) | <.001 | <.001 | <.001 | <.001 | <.001 |
|  | N | 1054 | 1054 | 1054 | 1054 | 1054 |
