# Supplementary material for: Translation and cultural adaption of MacLeod Clark professional identity scale among Chinese therapy students
Source: PLoS One. 2025 Jan 28;20(1):e0318101. doi: 10.1371/journal.pone.0318101 (PMC11774393; doi:10.1371/journal.pone.0318101)
Supplement: S5 Table — (DOCX) [file pone.0318101.s008.docx]

**S5: Table Methodological quality assessment using COSMIN checklist**

| **Assessment** | **Yes** | **No** | **?/NA** |
| --- | --- | --- | --- |
| **Design requirements** |  |  |  |
| 1 Was the percentage of missing items given? |  | **√** |  |
| 2 Was there a description of how missing items were handled? |  | **√** |  |
| 3 Was the sample size included in the analysis adequate? | **√** |  |  |
| 4 Were both the original language in which the instrument was developed, and the language in which the instrument was translated described? | **√** |  |  |
| 5 Was the expertise of the people involved in the translation process adequately described? e.g. expertise in the disease(s) involved, expertise in the construct to be measured, expertise in both languages | **√** |  |  |
| 6 Did the translators work independently from each other? | **√** |  |  |
| 7 Were items translated forward and backward? | **√** |  |  |
| 8 Was there an adequate description of how differences between the original and translated versions were resolved? | **√** |  |  |
| 9 Was the translation reviewed by a committee (e.g. original developers)? | **√** |  |  |
| 10 Was the instrument pre-tested (e.g. cognitive interviews) to check interpretation, cultural relevance of the translation, and ease of comprehension? | **√** |  |  |
| 11 Was the sample used in the pre-test adequately described? | **√** |  |  |
| 12 Were the samples similar for all characteristics except language and/or cultural background? |  | **√** |  |
| 13 Were there any important flaws in the design or methods of the study? |  | **√** |  |
| 14 for CTT: Was confirmatory factor analysis performed? | **√** |  |  |
| 15 for IRT: Was differential item function (DIF) between language groups assessed? |  |  | **√** |
